# Suicide rates in the Slovak Republic in 2011–2020

**DOI:** 10.1007/s00127-023-02486-0

**Published:** 2023-05-11

**Authors:** Veronika Sivcova, Norbert Moravansky, Dorota Novakova, Alexandra Brazinova

**Affiliations:** 1 Faculty of Medicine, Comenius University Bratislava, Špitálska 24, 813 72 Bratislava, Slovak Republic; 2 Forensic.sk Institute of Forensic Medical Analyses, Expert Institution, Bratislava, Slovak Republic

**Keywords:** Slovak Republic, Suicide rates, Undetermined intent deaths, Epidemiology

## Abstract

**Purpose:**

Suicide is a serious public health problem leading to premature mortality. The aim of the present study is to describe and analyze the trends of suicide rates in the Slovak Republic in 2011–2020.

**Methods:**

Trends of age-standardized suicide rates were described and incidence rate ratios of suicide were analyzed by negative binomial regression. The age-standardized rates of death by the undetermined intent and its ratio to suicide rate were calculated.

**Results:**

The overall suicide rate for the Slovak population was 7.58 per 100,000. Age-standardized suicide rate had a declining trend in the reported period, from 10.24 in 2011 to 6.65 per 100,000 in 2020. The highest suicide rate was in the oldest age groups. The male to female ratio was 6.09. The most common method of suicide in the Slovak population was hanging. The age-standardized rate of deaths by undetermined intent increased from 15.72 in 2011 to 18.46 per 100,000 in 2020.

**Conclusion:**

We observed the overall declining annual suicide mortality trends in the Slovak Republic in 2011–2020. Further investigation is necessary to understand the exceptionally high undetermined intent mortality.

## Introduction

Suicide is an act of intentional self-harm leading to death, causing emotional burden in families that are left behind and contributing to premature mortality in the society. Deaths by suicide are to a large extent preventable. More than 60% of suicides are accounted for by mental illness [1]. Therefore, early detection and treatment of commonly present underlying mental disorder can significantly reduce suicidal thoughts or behavior. However, the stigma surrounding mental disorders is very often a barrier to seeking help [2].

Undoubtedly, many premature deaths would be avoided if suicide was prevented. Globally, 800,000 people die from suicide annually, which translates to one person intentionally ending their life every 40 s. Rates of suicide do not affect the whole population equally. The majority of suicides occurs in low- and middle-income countries [3, 4], and in developed countries men have suicide rates three times that of women. Furthermore, suicides are the second leading manner of death in young people, preceded only by traffic accidents. However, according to the World Health Organization (WHO) 2019 global health estimates, the total number of suicides exceeded the number of deaths by traffic accidents, HIV, malaria or breast cancer every year [5–7].

Globally, the most common suicide methods include hanging, poisoning and the use of firearms with large regional differences in their respective prevalence [1, 7]. Understanding the method of suicide is likely to have implications for suicide prevention to tailor education and policy approaches.

Population groups at the highest risk of dying by suicide must be identified in order to develop effective prevention strategies, a process which requires accurate and recent data [8]. However, obtaining valid data remains challenging. Even though the WHO member states prepare the mortality statistics according to the WHO specifications, death certification practices vary over time and between countries and regions. Moreover, as suicide-related deaths are stigmatized in almost all cultures, miscoding, misclassification and underreporting of suicides likely occur, although the extent to which this happens is unknown [9].

In cases of doubt, caused by the lack of information and the resulting uncertainty of the intention of death, the examining physician uses the “undetermined intent” code of the International Classification of Diseases, 10^th^ revision (ICD-10) [10]. A proportion of undetermined intent deaths may be misclassified suicide cases; suicide studies either include deaths by undetermined intent in suicide mortality [13] or they exclude them [14–16]. Therefore, a correction for the underreporting of suicides should be performed in scientific studies at least by looking at trends, while accounting for the undetermined intent codes [11, 12].

The Slovak Republic is a high-income country located in Central Europe. Suicide rate trends have previously been described for the period of 1993–2015, 1993 being the year the Slovak Republic declared its independence. Prior to 1993, the Slovak Republic was part of Czechoslovakia, a state union formed with today’s Czech Republic [17, 18]. In the study period the overall suicide rate for the Slovak Republic declined, but the male to female ratio of suicides was much higher than the global average (5.9:1 versus 1.8:1). This period also saw an increasing proportion of undetermined intent among all deaths caused by an external cause.

We investigated the suicide trends in the Slovak Republic in the 10-year period of 2011–2020, a period that includes the first year of the COVID-19 pandemic. We also considered the methods of suicide and the development of the trend in the number of deaths being classified as undetermined intent. The main objective of this study was to present current data on suicide mortality in the Slovak Republic and to explore the validity of data to provide an accurate picture.

## Methods

### Data source

Data used for this study were obtained from the cause of death database of the Statistical Office of the Slovak Republic (SO SR). All deaths in the Slovak Republic between the years 2011 and 2020 classified as suicide according to the ICD-10 (codes X60-84) and as undetermined intent (codes Y10-Y34, Y87.2) were selected. The data contained the information on the date of death, age at death, sex, cause of death and external cause of death.

The suicide-related deaths were categorized based on the method of suicide into the following categories: X600-699 Poisoning, X700-709 Hanging, X710-719 Drowning, X720-749 Firearm, X780-789 Sharp object, X800-819 Jumping— from a high place or in front of a moving object, Others. The category Others included deaths coded as X75, X76, X79, X82, X83, X84 — in each instance less than ten cases were recorded under each of these codes for the overall period.

Deaths with undetermined intent (codes Y10-34, Y87.2) were characterized using the ICD-10 as “[events] where available information is insufficient to enable a medical or legal authority to make a distinction between accident, self-harm and assault” [10].

### Data analysis

Yearly crude suicide rates per 100,000 population with 95% confidence intervals [95% CI] were calculated for the total population. Further, sex- and age-specific rates were calculated for males and females, for the age groups 0–9, 10–19, 20–29, 30–39, 40–49, 50–59, 60–69, 70–79, 80–89, 90 + and also for broader age categories 0–19, 20–39, 40–59, 60–79 and 80 + years. To assess the possible underreporting of suicides, the annual crude undetermined intent death rates per 100,000 population with 95% CI were calculated for the total population; sex- and age-specific rates were calculated for males and females and for the same age groups as the suicide rates.

The annual mid-year population estimates used for the calculation were provided by the SO SR. Direct age-standardized mortality rates for suicides and undetermined intent deaths were calculated for the total population and separately for males and females using the revised European standard population [19].

To compare the male and female suicide and undetermined intent death rates, incidence rate ratios (IRR) with 95% confidence intervals were calculated by negative binomial regression for individual age groups separately and in total.

Deaths according to specific methods of suicide categories were expressed as proportions for the overall reported period 2011–2020. Undetermined death rate to suicide death rate ratio was also calculated.

The level of statistical significance was set at alpha = 0.05. All statistical analyses were performed by software STATA version 16.1 (StataCorp LP, College Station, TX, USA).

## Results

From 1st January 2011 to 31st December 2020, a total of 4532 people died by suicide in the Slovak Republic, out of which 3865 (85.28%) were men and 667 (14.72%) were women. Almost 60% of the suicide deaths were in the 40–69 years age group. Mean age at the time of suicide for females was 53.15 (SD 16.44) and for males 49.96 (SD 16.85).

The numbers, proportions and rates of suicide and undetermined intent deaths for the study period are shown by age group in Table 1. The highest suicide rates were observed in the 80–89 years age group, and the rate of undetermined intent increases with age. In the 10-year study period, 7923 deaths were classified as undetermined intent, 61.58% in men and 38.42% in women. Over 50% of deaths by undetermined intent occurred among those aged 70 years and above.

**Table 1:** Suicides and undetermined intent deaths and rates by 10-year age groups in the Slovak Republic, 2011–2020

Age group	Suicides		Undetermined intent	
	*n* (%)	Rate* [95% CI]	*n* (%)	Rate* [95% CI]
0–9	1 (0.02)	0.02 [0.00–0.09]	40 (0.50)	0.64 [0.45–0.86]
10–19	110 (2.43)	1.79 [1.47–2.15]	89 (1.12)	1.44 [1.16–1.78]
20–29	463 (10.22)	5.70 [5.19–6.25]	307 (3.87)	3.78 [3.37–4.23]
30–39	680 (15.00)	7.04 [6.53–7.59]	530 (6.69)	5.49 [5.03–5.98]
40–49	830 (18.31)	9.53 [8.90–10.20]	721 (9.10)	8.28 [7.69–8.90]
50–59	1052 (23.21)	13.03 [12.25–13.84]	1073 (13.54)	13.29 [12.50–14.11]
60–69	754 (16.64)	10.80 [10.05–11.60]	1169 (14.75)	16.75 [15.80–17.74]
70–79	412 (9.09)	10.70 [9.69–11.78]	1210 (15.27)	31.42 [29.67–33.24]
80–89	205 (4.52)	12.40 [10.76–14.22]	1962 (24.76)	118.6 [113.50–124.00]
90 +	25 (0.55)	10.48 [6.79–15.48]	822 (10.37)	344.7 [321.60–369.10]
total	4532 (100.00)	7.58 [7.37–7.81]	7923 (100.00)	13.26 [12.97–13.56]

*CI* confidence interval

^*^Rate per 100.000

Table 2 shows the incidence rate ratios (IRR) of suicide and undetermined intent in men compared to women. Overall, the men have an IRR of 6.09 [95%CI 5.60–6.62]) for suicide. For the oldest age groups, the IRR is above 10 (10.42 for 80–89 years age group and 14.66 for 90 + years age group). The IRR of undetermined intent deaths of men compared to women is 1.68 [95% CI 1.60–1.76] for the total population. The highest undetermined intent deaths IRR is for the age groups 30–39 and 40–49 years (5.84 [95% CI 4.56–7.57] and 5.05 [95% CI 4.14–6.22], respectively).

**Table 2:** Incidence rate ratios of suicides and undetermined intent deaths for men compared to women (male to female ratio), Slovak Republic, 2011–2020

Age group	Suicides IRR [95% CI]		UDI IRR [95% CI]	
0–9	0		1.76	[0.89–3.66]
10–19	3.80***	[2.36–6.37]	3.50***	[2.08–6.15]
20–29	8.10***	[6.01–11.12]	4.81***	[3.55–6.63]
30–39	8.26***	[6.44–10.74]	5.84***	[4.56–7.57]
40–49	6.62***	[5.39–8.19]	5.05***	[4.14–6.22]
50–59	5.30***	[4.50–6.28]	4.81***	[4.11–5.66]
60–69	5.22***	[4.34–6.32]	4.35***	[3.78–5.03]
70–79	7.96***	[6.12–10.49]	2.39***	[2.13–2.69]
80–89	10.42***	[7.23–15.38]	1.25***	[1.14–1.37]
90 +	14.66***	[4.95–58.74]	1.07	[0.91–1.25]
total	6.09***	[5.60–6.62]	1.68**	[1.60–1.76]

*IRR* incidence rate ratio, *UDI* undetermined intent

^***^p < 0.0001

^**^p < 0.01

**Fig. 1:**
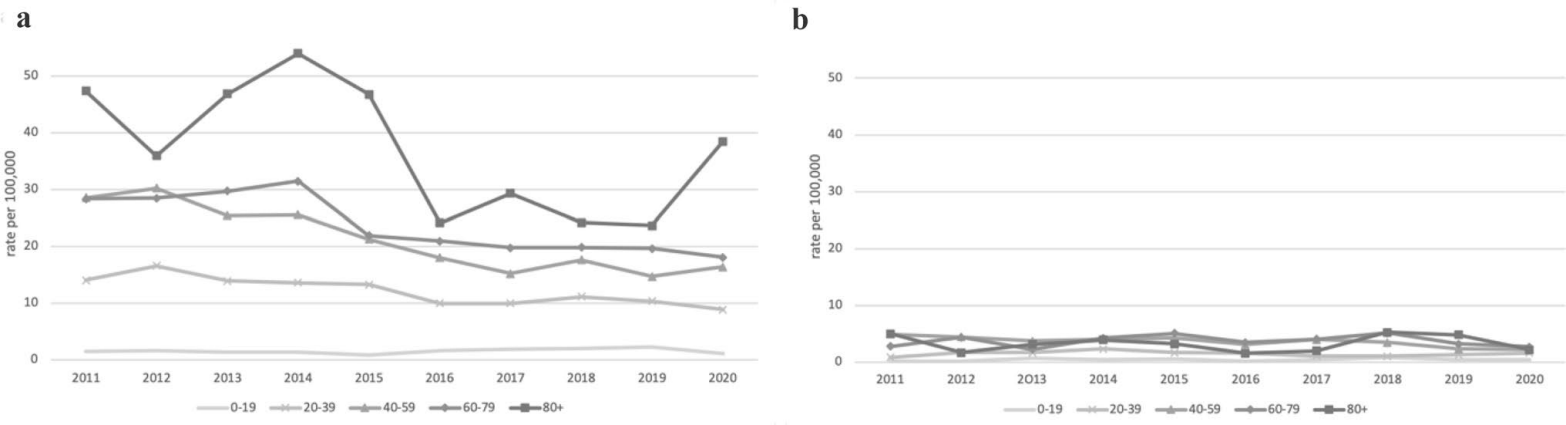
Age-specific suicide rates for men (**a**) and women (**b**) in the Slovak Republic, 2011–2020

Figure 1 shows the age-specific suicide rates for males and females per 100,000 population in five age groups. We observe an overall downward trend in the rates for males from 19.92 per 100,000 in 2011 to 12.62 per 100,000 population in 2020. The overall trend for females is stable, staying at 2 per 100,000 for the reported period. The suicide rate for men increases with age, the highest being in the oldest age group, 80 years and older. The peak male age-specific suicide rate of 53.97 per 100,000 population was observed in this age group in 2014. Rates of female suicide were highest among the 60–79 years age group, followed by the 80 + and 40–59 years age groups.

**Fig. 2:**
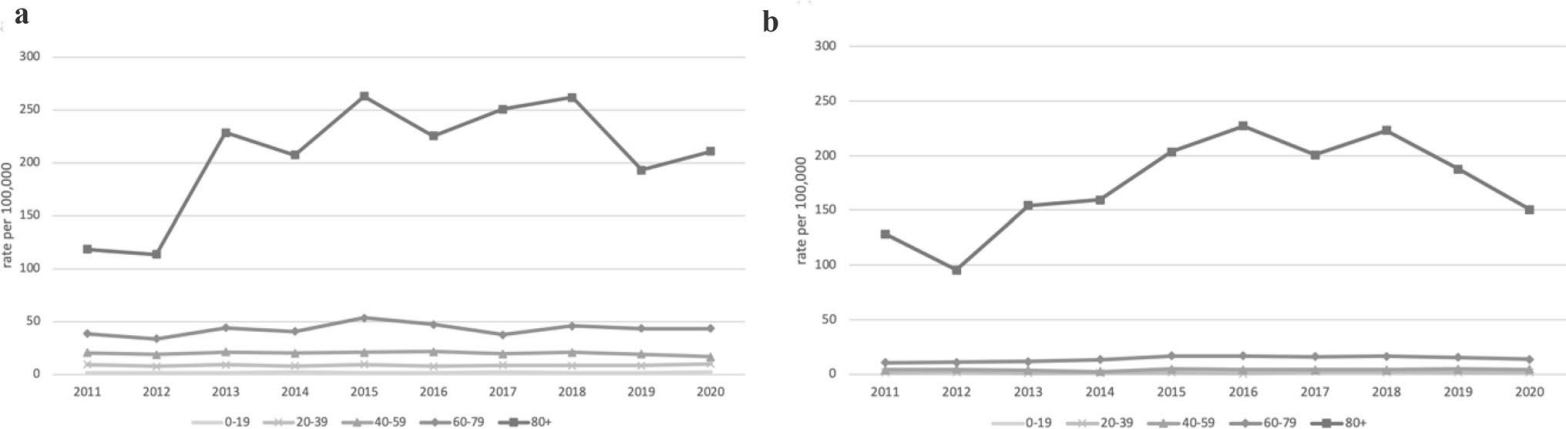
Age-specific undetermined intent death rates for men (**a**) and women (**b**) in the Slovak Republic, 2011–2020

To account for a possible underreporting of suicides we have described the deaths classified as having undetermined intent. These rates are several times higher than suicide rates. The highest rates are in the oldest age group (80 + years), for both men and women (Fig. 2). Rates for men in this age group increased from 118.35 per 100,000 population in 2011 to 210.74 per 100,000 population in 2020. For women the rates increased from 128.63 in 2011 to 150.42 per 100,000 population in 2020.

**Fig. 3:**
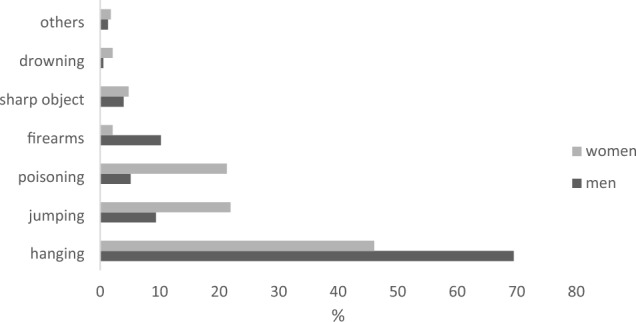
Proportions of suicide methods, by sex, 2011–2020, Slovak Republic

Figure 3 displays the methods of suicide for both men and women. The most common method was hanging; it was used in 69.47% of all male suicides and in 46.03% of all female suicides. The second most common method among men was firearms (10.22%), followed by jumping from high places or in front of a moving object (9.36%). For women, the second most common method was jumping (21.89%), followed closely by poisoning (21.29%).

**Fig. 4:**
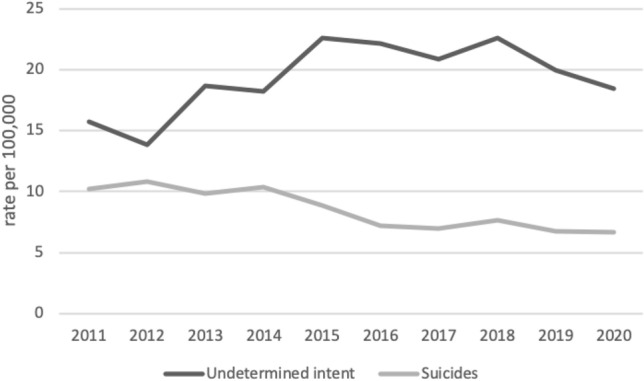
Age-standardized death rates classified as suicides and as undetermined intent in the Slovak Republic, 2011–2020

In the study period, we see a decline in suicide death rates overall, but an increase in the rate of deaths classified as undetermined intent for the total population (Fig. 4). The rate of age-standardized deaths by undetermined intent increased from 15.72 per 100,000 in 2011 to 18.46 per 100,000 in 2020. The age-standardized suicide rate decreased from 10.24 in 2011 to 6.65 in 2020.

The rate ratio of undetermined intent death rate to suicide rate was 1.53 in 2011 and 2.77 in 2020.

## Discussion

In our study, we have observed a decline in overall suicide mortality in the Slovak Republic in the 10-year period of 2011–2020. The highest suicide mortality rates were in the oldest male group. The same observation was made by the systematic analysis of suicides for the Global Burden of Disease Study 2016 (GBD 2016) across all regions and 195 participating countries. The age-standardized suicide mortality rates of the Central Europe region reported in GBD 2016 (13.0, 22.60 and 4.20 per 100,000, for total population, males and females, respectively) are higher than the age-standardized rates observed in Slovakia in 2016 (7.21, 13.32 and 2.09 per 100,000, for total population, males and females, respectively). The rates reported in Eastern European countries are even higher (27.50, 50.0, 8.30 for total population, males and females, respectively) [14]. Just as in other countries of Eastern and Central Europe, our results also show comparatively high rates for adults of working age. For example, the suicide rate reported for men aged 45–54 years old in Lithuania in 2019 was 50.07 per 100,000 [20]; in Slovakia for males of the same age group in 2019, it was 13.28 per 100,000 (data not shown in the article). The suicide mortality trend in Slovakia has a similar pattern to that of other Central and Eastern European countries, but the age-specific rates are much lower.

The exception to the overall downward trend in suicide mortality in the observed period was among men in the oldest age group during 2019 and 2020, where rates increased. This increase cannot be attributed to the COVID-19 pandemic as that started in the Slovak Republic in March 2020. There has been an overall decline in rates of suicide globally in recent decades. Still, the Slovak Republic belongs to the countries where suicide is among the top 10 leading manners of death [14]. Suicide mortality rate increases with age; however, among Slovak women, the rate of 40- to 79-year-olds exceeded the rate of the oldest age group 80 years and older during the period 2014–2017.

Higher suicide rates among older age groups can be attributed to several factors: social isolation, financial problems, psychiatric disorders (most commonly depression), physical illness, chronic pain and cognitive decline. These factors often accumulate with age and interact. Older men have significantly higher suicide rates across Europe, as well as in the USA (in particular, older white men) and Australia, compared to women [21].

Differences in suicide mortality by sex vary greatly across the world, with overall rates among men more than double those of women. The vast difference between male and female suicide rates is driven in part by social norms and attitudes regarding appropriate roles and responsibilities for men. Men who lose independence, social connectedness, or the ability to provide for themselves and their families are at greater risk of suicide than women, which is compounded by the fact that they are less likely to reach out for help [22]. Other contributing factors for high male suicide rates are higher rates of excessive alcohol consumption and the choice of more lethal methods for ending life, as compared to women [23].

The male to female incidence rate ratio for the whole population of Slovakia is 6.09 and increases with age; the greatest difference is in the oldest age group of 90 years and older, 14.66. The male to female ratios in the countries of Western Europe range from two to three, while in the post-communist countries the range is from five to seven [24]. Globally, the male to female ratio is the smallest in several developing countries, such as Liberia (where female suicide exceeds the rate of male suicide), Pakistan and Morocco (where the suicide rates of males and females are almost equal) [14].

Great global variability of suicide rates has many drivers. Studies indicate that those most frequently associated with suicide are economic crises; sociodemographic, sociocultural and religious factors; economic development; exposure to alcohol and drugs; family history of mental illness; and access to suicide means [25]. There is also considerable international variability in the methods of suicide. Hanging or strangulation is universally available, which makes it the most common method of suicide in many countries, including the Slovak Republic. It was used as a means of death by 69.47% of men and 46.03% of women. Self-poisoning ranked as the second highest suicide method for both males and females in European countries [26, 27]. The second most common method observed in our study was jumping from a high place or in front of a moving object. Self-poisoning was the third most common means of suicide for Slovak women (21.29%) and the fourth for men (5.12%). The third most common method for men was death by firearm (10.22%).

The validity of suicide statistics is greatly affected by the cause of death ascertainment procedures that vary across countries. At the same time, stigma related to suicide deaths prevails globally. Some states still list attempted suicide as a crime in their criminal statutes. All these factors contribute to a certain degree of misclassification of suicide. Suicide researchers hypothesize that a proportion of suicides is hidden within the undetermined intent category; however, the extent is very difficult to quantify [28].

The finding that suicide rates in the Slovak Republic are lower than in other countries in Central Europe needs to be interpreted with caution. In the reported period, we have observed an increasing trend in the undetermined intent mortality in the Slovak Republic, with a rate 13.26 per 100,000 in 2020. This is consistent with the trend observed in our previous study, where we described suicide and undetermined mortality since the establishment of the independent Slovak Republic in 1993 [13]. The highest rate of deaths by undetermined intent was observed in the oldest age group (80 years old and older) and it is similar for both men and women. Half of all deaths classified as undetermined intent were among people 70 years of age and above. This is the population subgroup that has the most deaths classified as undetermined intent. Värnik et al. reported an average undetermined death (UD) rate for 15 countries of European Union (EU-15) of 1.97 per 100,000 (the Slovak Republic exceeds this by almost seven times), and the ratio of UD rate to suicide rate of 0.19 [29]. According to our study, the age-standardized UD rate/suicide rate ratio in the Slovak Republic in 2012 was about 1.54 and in 2020 increased to 2.78. To our knowledge, in no other country in Europe does the UD rate exceeds the suicide rate. Värnik et al. proposed a benchmark of UD rate/suicide rate ratio of 0.2 (20%) for Europe. Such a benchmark would help in evaluating the quality of suicide statistics. Another indicator proposed in the same study is 2.0 UD cases per 100,000. The Slovak Republic massively exceeds these proposed benchmarks.

Undetermined intent as the manner of death is given by the physicians completing death certificates and by forensic pathologists when they lack information about the case circumstances. We currently have no information on the reasons for such a high rate of undetermined intent mortality in the Slovak Republic. Reasons for such classification may include insufficient information provided by the police from the case investigation or insufficient training and performance of examining physicians in death certificate completion. The cause of death reporting procedure in the Slovak Republic follows international guidelines set by the World Health Organization. The reasons for high rate of undetermined intent mortality in Slovakia need to be studied in detail.

A strength of this study is the delivery of current information on the distribution of suicide mortality in the population of the Slovak Republic, a current gap.

The main limitation of the study is an unclear validity of suicide statistics. Some of the deaths classified as undetermined intent may be caused by suicide, however, the extent of possible misclassification is unknown.

The main implication of this study is the need for a detailed investigation of the cause of death ascertainment procedures in the Slovak Republic, with a special emphasis on the classification of the death as having undetermined intent. The finding of the highest and increasing suicide mortality rate among older males provides information, on which targeted suicide prevention in this population group can be based.

## Conclusion

We have investigated the trends of suicide mortality, methods of suicide and mortality by undetermined intent in the Slovak Republic in the period 2011–2020. Despite the overall declining annual suicide mortality trends, further investigation is needed to understand the exceptionally high numbers of deaths being classified as undetermined intent.

## References

[1] Ferrari AJ, Norman RE, Freedman G, Baxter AJ, Pirkis JE, Harris MG (2014). The burden attributable to mental and substance use disorders as risk factors for suicide: findings from the Global Burden of Disease Study 2010. PLoS ONE.

[2] Wei Y, McGrath P, Hayden J, Kutcher S (2018). The quality of mental health literacy measurement tools evaluating the stigma of mental illness: a systematic review. Epidemiol Psychiatr Sci..

[3] Bachmann S (2018). Epidemiology of suicide and the psychiatric perspective. Int J Environ Res Public Health.

[4] Collaborators GBDCoD (2018). Global, regional, and national age-sex-specific mortality for 282 causes of death in 195 countries and territories, 1980–2017: a systematic analysis for the Global Burden of Disease Study 2017. Lancet.

[5] Organization WH. Suicide in the world. Global health estimates. 2019.

[6] Mojahedi M, Esmaeili A, Mahdizadeh K, Nakhaei MH, Salehiniya H, Sahranavard S (2021). Trends of suicide attempts and factors related to completed suicide during the years 2014–2019 in South Khorasan province. Iran. Asian J Psychiatr..

[7] Etzersdorfer E, Voracek M, Kapusta N, Sonneck G (2005). Epidemiology of suicide in Austria 1990–2000: general decrease, but increased suicide risk for old men. Wien Klin Wochenschr.

[8] Volker D, Zijlstra-Vlasveld MC, Brouwers EP, van Lomwel AG, van der Feltz-Cornelis CM (2015). Return-to-work self-efficacy and actual return to work among long-term sick-listed employees. J Occup Rehabil.

[9] Bertolote JM, Fleischmann A (2002). Suicide and psychiatric diagnosis: a worldwide perspective. World Psychiatry.

[10] World Health Organization (1993) The ICD-10 classification of mental and behavioural disorders: diagnostic criteria for research. Geneva: World Health Organization. xiii:248.

[11] Bjorkenstam C, Johansson LA, Nordstrom P, Thiblin I, Fugelstad A, Hallqvist J (2014). Suicide or undetermined intent? A register-based study of signs of misclassification. Popul Health Metr.

[12] Mohler B, Earls F (2001). Trends in adolescent suicide: misclassification bias?. Am J Public Health.

[13] HMG/DH. Preventing suicide in England: a cross-government outcomes strategy to save lives 2012. Available from: https://assets.publishing.service.gov.uk/government/uploads/system/uploads/attachment_data/file/430720/Preventing-Suicide-.pdf. Accessed 20 Feb 2023

[14] Naghavi M, Global Burden of Disease Self-Harm C (2019). Global, regional, and national burden of suicide mortality 1990 to 2016: systematic analysis for the Global Burden of Disease Study 2016. BMJ.

[15] Maheen H, Taouk Y, LaMontagne AD, Spittal M, King T (2022). Suicide trends among Australian construction workers during years 2001–2019. Sci Rep.

[16] Boricevic Marsanic V, Silobrcic Radic M, Flander TM (2022). Trends in adolescent completed suicide in croatia for the period of 2000 to 2020. Psychiatr Danub.

[17] Brazinova A, Moravansky N, Gulis G, Skodacek I (2017). Suicide rate trends in the Slovak Republic in 1993–2015. Int J Soc Psychiatry.

[18] Brazinova A, Majdan M (2016). Road traffic mortality in the Slovak Republic in 1996–2014. Traffic Inj Prev.

[19] European Commission, Eurostat, Pace, M., Gissler, M., Lanzieri, G., et al., Revision of the European Standard Population : report of Eurostat's task force : 2013 edition, Publications Office, 2013. https://data.europa.eu/doi/10.2785/11470

[20] Lange S, Rehm J, Tran A, Bagge CL, Jasilionis D, Kaplan MS (2022). Comparing gender-specific suicide mortality rate trends in the United States and Lithuania, 1990–2019: putting one of the "deaths of despair" into perspective. BMC Psychiatry.

[21] King K, Dow B, Keogh L, Feldman P, Milner A, Pierce D (2020). "Is life worth living?": The role of masculinity in the way men aged over 80 talk about living, dying, and suicide. Am J Mens Health.

[22] Houle J, Mishara BL, Chagnon F (2008). An empirical test of a mediation model of the impact of the traditional male gender role on suicidal behavior in men. J Affect Disord.

[23] Cibis A, Mergl R, Bramesfeld A, Althaus D, Niklewski G, Schmidtke A (2012). Preference of lethal methods is not the only cause for higher suicide rates in males. J Affect Disord.

[24] Pray L, Cohen C, Mäkinen IH, Värnik A, MacKellar FL (2013) Suicide in Eastern Europe, the Commonwealth of Independent States, and the Baltic Countries: Social and Public Health Determinants. IIASA Research Report. IIASA, Laxenburg, Austria: RR-13-001

[25] Sinyor M, Tse R, Pirkis J (2017). Global trends in suicide epidemiology. Curr Opin Psychiatry.

[26] Ajdacic-Gross V, Weiss MG, Ring M, Hepp U, Bopp M, Gutzwiller F (2008). Methods of suicide: international suicide patterns derived from the WHO mortality database. Bull World Health Organ.

[27] Varnik A, Kolves K, van der Feltz-Cornelis CM, Marusic A, Oskarsson H, Palmer A (2008). Suicide methods in Europe: a gender-specific analysis of countries participating in the "European alliance against depression". J Epidemiol Community Health.

[28] Kapusta ND, Tran US, Rockett IR, De Leo D, Naylor CP, Niederkrotenthaler T (2011). Declining autopsy rates and suicide misclassification: a cross-national analysis of 35 countries. Arch Gen Psychiatry.

[29] Varnik P, Sisask M, Varnik A, Arensman E, Van Audenhove C, van der Feltz-Cornelis CM (2012). Validity of suicide statistics in Europe in relation to undetermined deaths: developing the 2–20 benchmark. Inj Prev.

